# Keeping the Teeth Dry

**Published:** 1868-03

**Authors:** H. Scott

**Affiliations:** Lancaster, O.


### KEEPING THE TEETH DRY.

BY H. SCOTT.

I was present but a few minutes on Wednesday afternoon, when the late Convention of the Mississippi Valley Dental Association had under consideration the "*rubber dam*," etc., etc. I desired to say something to the meeting on that subject, on the following day, but an unexpected exigency in my business required me to leave the city on Thursday morning.

I have passed through the era of appliances for keeping the tongue out of the way, and the saliva from the teeth to be filled, and have endured my full share of fretting over the troubles they have given me. I pay now no attention to any of them. My methods are original, simple; and to me, entirely satisfactory. I am able to keep my nerves tranquil during the process of even difficult operations. This is every thing with me; for I hold that no man can accomplish much for good, in any thing, when his equilibrium is unsettled.

Of course our perplexities arise mostly when working on the inferior teeth. My method is this. When the cavities are ready for the filling, I require the patient to turn the head on the opposite side from the tooth to be filled. Under the depending corner of the mouth I place a folded napkin, (I prefer soft linen on account of its better absorbing qualities,) and tell the patient to give no attention to the saliva, but allow it to overflow, as the linen will take it all up. I then request the patient to close the opposite hand, except the index finger. Then with the closed fist carried round on the cheek, the free finger is pushed to the top and edge of the tongue opposite the work to be done, and that organ pressed downward and towards the other side of the mouth. I then introduce the forefinger of my left hand under the cheek, and press it away from the dental arch, thus leaving the teeth lifted up high and dry, and quite above high water mark. In this favorable position of things, and with the strict injunction not to move or change a muscle, I can work in the dry an hour, if I choose. At least I frequently conduct a somewhat tedious operation from beginning to end, with entire success, and not the least interruption from overflowing. Before placing things in position, as above, I have every thing at hand; and with my left finger on duty, I do all the manipulation with my right hand. And whether I employ the mallet, or do hand work, it is all pleasant and easy. In fang filling, or large and difficult cavities, I stop and begin again as many times as the ease of my patient requires, always assuming the same position, and drying out as at first.

There is another result of this method, that has pleased me very much. I have not only not found a single person to object, but, on the contrary, I am sure that every one has been more than willing to render the service asked of them; they feel assured. They feel that they are helping themselves, and protecting themselves, to some extent, against danger, a sense of which it is almost impossible to avoid. At least they do not feel so utterly abandoned, for the time being, to the merciless assaults that is being made upon their sensibilities.

But I have occasionally fell upon a case, and so has every operator, where neither this, nor any other mild method would amount to anything. When I get in hand a patient that can not, and will not keep the lips, chin, or any part of the head in any required position over two or three seconds at one time, I quit; I let go every thing. It does no good to scold, coax, or prompt; it is all time and words wasted. I do not, under such circumstances, even let the patient know that any thing is wrong; for I have long since learned that to conduct an operation to even respectable success, the nerves of both parties must be in their normal state.

After a short respite I take hold of my work and fill the cavity, submarine; because it is the only way it can be done. And here I take occasion to say that I have filled lower teeth more than twenty years ago, under water, that are good to-day; apparently as good as those filled at the same time in the dry. It is not so difficult to do; but one must keep cool, and work with deliberation. Firm borders; good retaining points; and the metal placed in position as solidly as the force used can make it, is good work, whether done in the wet or in the dry; provided always, that no possible crevice be left any where for the admission of fluids behind the filling.

The reader will see that my special point has been to tell how I manage to keep the lower teeth dry while I am filling them. I hope I shall help some who are perplexed *now*, as I *have* been.

Lancaster, O.

